# Inflammatory markers before and after farrowing in healthy sows and in sows affected with postpartum dysgalactia syndrome

**DOI:** 10.1186/s12917-018-1382-7

**Published:** 2018-03-12

**Authors:** Marianne Kaiser, Magdalena Jacobson, Pia Haubro Andersen, Poul Bækbo, José Joaquin Cerón, Jan Dahl, Damián Escribano, Stine Jacobsen

**Affiliations:** 1 Department of Veterinary Clinical Sciences, Faculty of Health and Medical Sciences, Copenhagen University, Højbakkegård Alle 5, 2630 Taastrup, Denmark; 2 Department of Clinical Sciences, Faculty of Veterinary Medicine and Animal Science, Swedish University of Agricultural Sciences, p.o. Box 7054, SE-750 07 Uppsala, Sweden; 3 SEGES, Danish Pig Research Center, Danish Agriculture & Food Council, Agro Food Park 15, 8200 Aarhus N, Denmark; 4 Department of Animal Medicine and Surgery, Regional “Campus of Excellence Mare Nostrum”, University of Murcia, 30100 Espinardo, Murcia, Spain; 5 Danish Agriculture and Food Council, Axelborg, Axeltorv 3, 1709 Copenhagen V, Denmark

**Keywords:** PDS, Inflammatory markers, Postpartum, Dysgalactia, Sow

## Abstract

**Background:**

The pathogenesis of postpartum dysgalactia syndrome (PDS) in sows is not fully elucidated and affected sows often present vague clinical signs. Accurate and timely diagnosis is difficult, and PDS is often recognized with a delay once piglets begin to starve. Increased rectal temperature of the sow is an important diagnostic parameter, but it may also be influenced by a number of other parameters and is thus difficult to interpret. Inflammatory markers may be important adjuncts to the clinical assessment of sows with PDS, but such markers have only been studied to a limited extent. The objective was to characterize the inflammatory response in healthy sows and in sows suffering from PDS, and to identify biomarkers that may assist in early identification of PDS-affected sows.

**Results:**

Thirty-eight PDS-affected (PDS+) and 38 healthy (PDS-) sows underwent clinical examination and blood sampling every 24 h, from 60 h before the first piglet was born to 36 h after parturition. In both groups, inflammatory markers changed in relation to parturition. Most inflammatory markers changed 12-36 h after parturition [white blood cell counts (WBC), neutrophil counts, lymphocyte counts, tumor necrosis factor alpha (TNF-α), interleukin 6 (IL-6), serum amyloid A (SAA), C-reactive protein (CRP), haptoglobin (Hp), iron (Fe) and albumin (ALB)]. Changes in neutrophil counts, lymphocyte counts, CRP, Fe and ALB were observed -12 to 0 h before parturition. WBC, neutrophil and lymphocyte counts, serum concentrations of TNF-α, IL-6, Hp and Fe differed between PDS+ and PDS- sows. These differences were mainly apparent 12 to 36 h after parturition, but already at 12 h before parturition, PDS+ sows had lower lymphocyte counts than PDS- sows.

**Conclusions:**

Parturition itself caused significant inflammatory changes, but PDS+ sows showed a more severe response than PDS- sows. WBC, neutrophil and lymphocyte counts, and concentrations of TNF-α, IL-6, Hp and Fe can be potential biomarkers for PDS. Lymphocyte counts may be used to detect PDS at pre-partum. To assess their diagnostic potential, these markers must be investigated further and most likely combined with assessment of clinical parameters and other biomarkers for improved identification of sows at risk of developing PDS.

**Electronic supplementary material:**

The online version of this article (10.1186/s12917-018-1382-7) contains supplementary material, which is available to authorized users.

## Background

Postpartum dysgalactia syndrome (PDS) is a common disease in sows with recently reported prevalences varying from 6.0 to 48.2% [1–4]. The pathogenesis of PDS is complex and not fully elucidated. Mastitis, metritis and agalactia syndrome (MMA) and coliform mastitis may all cause dysgalactia, but these disease complexes are considered to be the more readily observable components of the PDS complex [5, 6]. Therefore, a subgroup of sub-clinically affected sows, which are difficult to detect in the early stages of the disease, seems to occur. These cases of PDS may only be discovered once piglets start to lose weight and display milk-searching behaviour. Mortality increases when colostrum intake during the first 24 h of life is less than 200 g per piglet [7], and increased piglet mortality among MMA-affected litters has been reported [8]. Furthermore, increased prevalence of stillborn piglets has been found in litters from sows with fever or elevated rectal temperature before [9] and after farrowing [10]. The impact of PDS on the welfare of sows and piglets is thus substantial.

Currently, elevated rectal temperature seems to be one of the more reliable indicators of PDS [4, 11, 12], but as rectal temperature is influenced by metabolic status, parity, circadian rhythm and days postpartum (p.p.), interpretation of the measured rectal temperature is not straight forward [4]. A threshold temperature of 39.5°C is most often applied as a cut-off value when used for detection of PDS [11, 13–20]. Other clinical signs, e.g. anorexia, inflamed udder, decreased demeanor, and milk-searching behavior among piglets, have been used in conjunction with rectal temperatures in an attempt to identify sows with PDS [13, 18, 19, 21, 22].

Systemic inflammation in sows with PDS has only been investigated to a limited degree. Increased blood levels of haptoglobin (Hp) [8, 23], tumor necrosis factor alpha (TNF-α) and interleukin-6 (IL-6) [24] have been found in sows diagnosed with MMA, and a decrease in white blood cell counts (WBC) and neutropenia was observed in sows developing agalactia after experimental intramammary infusion of *Escherichia (E.) coli* [25]. Furthermore, increases in concentrations of cytokines and acute phase proteins (APPs) have been demonstrated in sows with experimental mastitis elicited by intramammary infusion of *E. coli* [26] or lipopolysaccharide (LPS) [27].

Detection of PDS remains elusive, and it is thus possible that the prevalence of sub-clinically affected sows is significant. Consequently, the diagnosis may be delayed until the piglets start expressing hunger, at which time the disease has severe consequences on animal welfare and production. Early detection of the disease (most preferably before or during parturition, or at least before piglets show signs of starvation) is thus desirable and may be aided by assessment of biomarkers in blood. The objective of our study was to characterize the periparturient inflammatory response in healthy sows and in sows suffering from PDS with the goal of identifying biomarkers that may assist in identification of affected animals early in the course of disease.

## Methods

### Herd and animals

A case-cohort study was performed from March 2014 to November 2014, including 38 PDS affected (PDS+) and 38 healthy (PDS-) multiparous sows. Samples were collected from a Danish sow herd within the Danish Specific Pathogen-Free (SPF) system. The farm was chosen based on a treatment rate of at least 20% of all farrowing sows. Usually, 20–30% of the sows would receive medical treatment in the periparturient period. The sows were housed in confined crates from 1 week before farrowing until 3 weeks after farrowing. Between each batch of sows, the farrowing units were cleaned and disinfected. The farrowing pens had partly slatted floors with 2/3 solid concrete and 1/3 iron bars measuring 1.6 × 2.6 m^2^. The sows were fed 4 times per day with liquid feed and assigned straw according to the Danish law of animal welfare. All sows were of the Danish cross-breed (Landrace/Yorkshire).

### Experimental design

According to the following and assumptions of the syndrome [28], a broad clinical definition of PDS was adopted. PDS+ sows would have at least two of the following clinically visible characteristics: 1. anorexia, defined as “trough not empty 30 minutes after feeding”, 2. inflammation of the udder, characterized by redness, swelling and increased skin temperature, 3. rectal temperature ≥ 39.5°C.

From each of 9 batches, approximately 12 sows were randomly selected and monitored when they entered the farrowing unit. All sows (*n* = 109) were sampled every 24 h from 60 h before expected parturition and to a maximum of 36 h p.p. or until PDS occurred. For ethical reasons, sows were treated as described below and precluded from further sampling as soon as PDS was detected. In the following order, monitoring prior to parturition included: 1. samplings of saliva and ear venous blood before morning feeding (these data were used for other research purposes and are not shown), 2. veterinary clinical assessment after feeding with recording of the general demeanor, appetite, rectal temperature, respiration frequency, capillary re-filling time, skin color, eye mucosa color, vaginal mucosa color, vaginal discharges, fecal consistency, and signs of inflammation (subjective assessment of skin temperature by palpation, capillary refill time, and hyperemia) of the mammary glands, 3. blood sampling from *v. jugularis* as described below, and 4. blood sampling from *v. epigastrica caudalis superficialis* [*v. mammaria cranialis*] (these data were used for other research purposes and are not shown). After parturition, a morning and an afternoon milking was included in the procedure for other research purposes.

Sows categorized as PDS+ were administered medical treatment by the farmer immediately after the clinical examination and sample collection. The medical treatment consisted of systemic antibiotics, either 10.000 IU/kg bw of benzyl procaine penicillin (Noropen® vet., ScanVet, Denmark) or 16 mg/kg bw of trimethoprim-sulfadiazin (Norodine® vet., ScanVet, Denmark), and 0.4 mg/kg bw of meloxicam (Loxicom®, ScanVet, Denmark). Sows that farrowed prematurely and sows treated for reasons other than PDS were excluded from the study.

Eventually, 38 sows were defined as PDS+ sows, and these were retrospectively matched with 38 PDS- sows. The match was done in the following descending order of importance: 1. batch, 2. parity, and 3. date of parturition. Data from the remaining 33 sows were not included in the study.

### Sampling

Blood samples were collected from *v. jugularis* in tubes with no additive for preparation of serum (BD, New Jersey, US) and with EDTA for haematological analyses (BD, New Jersey, US). To reduce stress and build up confidence, the sows were fed small sugar cubes after handling. All blood samples were kept at room temperature for a maximum of 30 minutes before being processed. The additive-free tubes were centrifuged for 10 min. at 3,000 × g and the serum was separated and stored at – 80°C until analysis, which was performed within 15 months from first sampling date. The EDTA tubes were carefully mixed and blood smears prepared within 2 h. Blood smears underwent cytological examination. Blood samples were stored at 5°C for a maximum of 48 hours before they were shipped to the Veterinary Diagnostic Laboratory at University of Copenhagen for determination of WBC count, hematocrit (Ht), hemoglobin (Hb), iron (Fe), Albumin (ALB) and total protein (TP), which were performed by the Hematology System Complete Blood Count method using an automated biochemistry analyzer (ADIVA 2120/2120i, Siemens Healthcare A/S, Denmark). Blood smears were stained with modified Wright stain (Siemens AG, Germany), and differential count of WBC was performed as described [29]*.* Concentrations of the cytokines interleukin-1 (IL-1), IL-6 and TNF-α in serum were determined by commercially available pig-specific ELISAs (Porcine IL-1 beta/IL-1F2 Quantikine ELISA Kit, Porcine IL-6 Quantikine ELISA Kit, and Porcine TNF-alpha Quantikine ELISA Kit, all from R&D Systems, Minneapolis, USA) as described previously [30]. The absorbance was read at 450 nm using a microtiter plate reader (Multiskan EX, Thermo LabSystems, Massachusetts, USA). Concentrations below the detection limit of the assay were set at the detection limit for the assay in question in the calculations (TNF-α = 23.40 pg/mL, IL-1 = 39.10 pg/mL, IL-6 = 18.80 pg/mL). Concentrations in serum of the acute phase protein serum amyloid A (SAA) was determined by a previously described commercial multispecies SAA ELISA kit (Tridelta Development Ltd., Ireland) according to the manufacturer’s instructions [31]. The absorbance was read at 450 nm using a microtiter plate reader (BIO-TEK, Vermont, US). Hp and C-reactive protein (CRP) levels were measured in serum using an automated biochemistry analyzer (Olympus AU600 Automatic Chemistry Analyzer, Olympus Europe GmbH, Germany) with commercial quantitative turbidimetric tests produced by SPINREACT, S.A.U (Spain) and Beckman Coulter® (California, USA), respectively. Assays for Hp and CRP were performed as reported before [32]. The APP assays all had intra-run and inter-run coefficients of variation < 10%, and the limits of detection were 10 mg/L for Hp, 0.6 mg/L for CRP and 3.06 mg/L for SAA. Where concentrations were higher than the upper limit of the assay, concentrations were set at the concentrations of the highest calibrator (SAA, 500 mg/L, *n* = 7 samples).

### Statistical analyses

Retrospectively, the exact sampling times (date:hour:min.) were determined relative to the exact time of farrowing of the first piglet (date:hour:min.) which was recorded by videos. Sampling times were grouped into time intervals where 0 h was the parturition time of the first piglet: A. -60 to -36 h; B. -36 to -24 h; C. -24 to -12 h; D. -12 to 0 h; E. 0 to 12 h; F. 12 to 24 h, and G. 24 to 36 h. The number of observations (*n*) within each interval varied because of variation in the individual sampling times relative to parturition (0 h) [For illustrative explanation, see Additional file 1].

For statistic evaluation, two autoregressive linear regression models (A and B) were performed in the PROC MIXED procedure of Statistic Analytical Software, Enterprise Guide 7.1 (SAS® Institute, Cary, North Carolina, USA). Least-squares means (LSMEANS) and standard deviation (SD) were included in the statistic model A. Model A was OUTCOME PARAMETER_ij_ = μ + TIME_i_ + GROUP_j_ + TIME*GROUP_ij_ + ε where OUTCOME PARAMETER_ij_ was the measured value of the inflammatory parameter, μ was the observations value at time 0, TIME_i_ was explanatory variable “time intervals A-G”, GROUP_j_ the explanatory variable “PDS+/PDS-”, TIME*GROUP_ij_ the interaction between the two groups and change over time and ε was the random residual error term. When significant interaction was identified using model A, differences between the relevant groups and time intervals were accepted. In case of non-significant interaction, model A were replaced with model B which was OUTCOME PARAMETER_ij_ = μ + TIME_i_ + GROUP_j_ + ε. If a non-significant change in TIME_i_ occur in model B, the OUTCOME PARAMETER_ij_ was considered non-significant. For significant TIME_i_ value, differences in the relevant groups were still accepted and recorded from the model A output. In case of a significant effect of GROUP_j_, an overall effect of group was accepted. Significance was considered if *p*<0.05. Parity and body condition score were included as explanatory variables. By preliminary analyses, obstetric aid and farrowing length were found not to be associated with any of the outcome variables. Natural logarithm was performed for lymphocytes, IL-6, TNF-α, CRP, SAA and TP because assumptions concerning residual plots and test for normality were unlikely.

## Results

### Clinical findings

The 38 sows categorized as PDS+ constituted 34.9% of the farrowings in the randomly selected 109 sows. PDS+ sows were on average treated 18.6 h (range 6-26.9 h) after the first piglet was born. Obstetric aid was performed in 18 PDS+ sows and 11 PDS- sows. Reduced appetite was observed in 10 PDS+ sows and 5 PDS- sows at 0-12 h, while 12 PDS+ sows and 12 PDS- showed reduced appetite at 12-24 h (with 1 sow having complete anorexia). Mean parity, relevant production results, and clinical findings are shown in Table 1.

**Table 1:** Parity and productions results for 38 sows with postpartum dysgalactia syndrome (PDS+) and 38 healthy sows (PDS-) together with key clinical parameters in time interval A, E and F. The number of observations (*n*) in each interval varies because of variation in individual sampling times relative to parturition (0 h) [For further explanation see Additional file 1]

Sow related observations	PDS+				PDS-			
	*n*	Mean	Min	Max	*n*	Mean	Min	Max
Parity and production								
Parity, no.	38	4.26	2	7	38	4.12	2	7
Liveborn, no.	37	16.9	10	23	38	17.3	6	24
Stillborn, no.	38	0.18	0	3	38	0.32	0	3
Clinical findings time point A (-60 to -36 h p.p.)								
Rectal temp, °C	18	38.1	37.4	38.7	22	38.2	37.6	38.8
Increased mammary skin temperature, no. of glands/sow	18	0.1	0	1	22	0.9	0	14
Heart rate, beats/min.	18	122	96	152	22	112	82	160
Respiratory rate, breaths/min.	18	46	22	86	22	45	22	88
Clinical findings time point E (0 to12 h p.p.)								
Rectal temp, °C	15	38.9	38.0	40.1	10	38.6	37.3	39.3
Increased mammary skin temperature, no. of glands/sow	14	4.4	0	16	10	1.5	0	15
Heart rate, beats/min.	14	112	68	144	10	117	92	144
Respiratory rate, breaths/min.	15	31	13	112	10	37	12	84
Clinical findings time point F (12 to 24 h p.p.)								
Rectal temp., °C	23	39.5	38.1	40.5	25	39.0	38.1	39.3
Increased mammary skin temperature, no. of glands/sow	23	10.4	0	17	24	6.0	0	16
Heart rate, beats/min.	23	117	84	158	25	101	74	144
Respiratory rate, breaths/min.	23	25	12	38	24	27	12	66

### Inflammatory markers

#### Change in level of inflammatory markers in relation to parturition

**Fig. 1:**
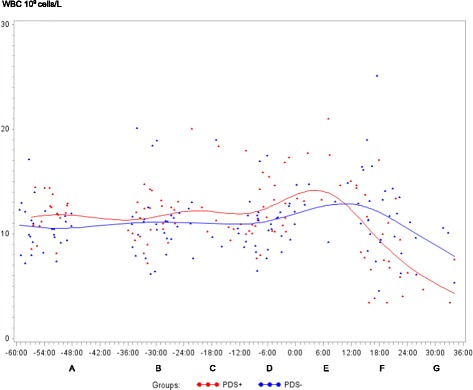
White blood cell (WBC) counts (10^9^ cells/L) in sows with postpartum dysgalactia syndrome (PDS+, red) and healthy sows (PDS-, blue) sampled from 60 h before until 36 h after parturition (time interval A-G). Each dot represents the exact sample time of each observation relative to the exact birth of the first piglet (0 h). The lines show the mean value (Normal range; 11.3-22.8 × 10^9^ cells/L)

**Fig. 2:**
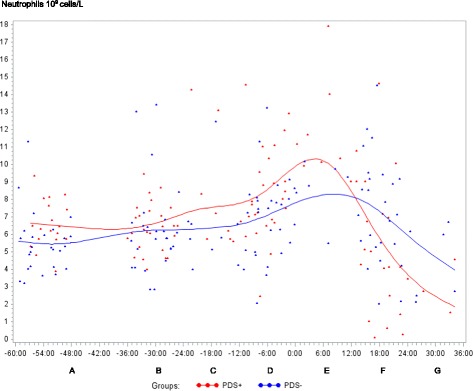
Neutrophils counts (10^9^ cells/L) in sows with postpartum dysgalactia syndrome (PDS+, red) and healthy sows (PDS-, blue) sampled from 60 h before until 36 h after parturition (time interval A-G). Each dot represents the exact sample time of each observation relative to the exact birth of the first piglet (0 h). The lines show the mean value (Normal range; 3.1-9.6 × 10^9^ cells/L)

**Fig. 3:**
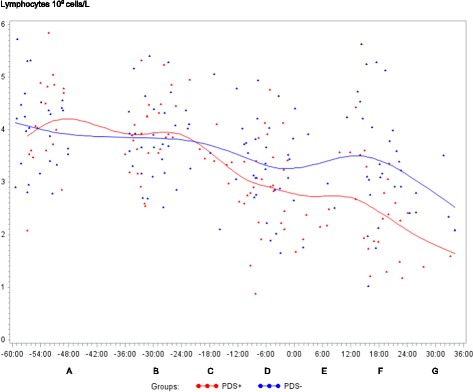
Lymphocyte counts (10^9^ cells/L) in sows with postpartum dysgalactia syndrome (PDS+, red) and healthy sows (PDS-, blue) sampled from 60 h before until 36 h after parturition (time interval A-G). Each dot represents the exact sample time of each observation relative to the exact birth of the first piglet (0 h). The lines show the mean value (Normal range; 4.6 – 10.0 × 10^9^ cells/L)

**Fig. 4:**
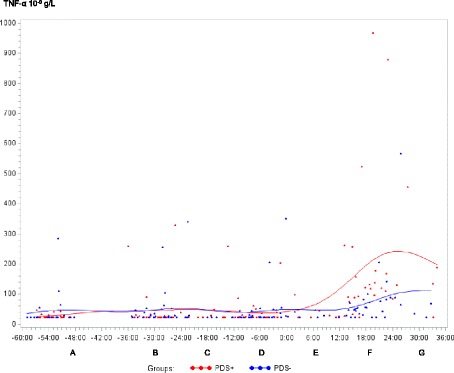
Tumor necrosis factor alpha (TNF-α) concentration (10^-9^g/L) in sows with postpartum dysgalactia syndrome (PDS+, red) and healthy sows (PDS-, blue) sampled from 60 h before until 36 h after parturition (time interval A-G). Each dot represents the exact sample time of each observation relative to the exact birth of the first piglet (0 h). The lines show the mean value

**Fig. 5:**
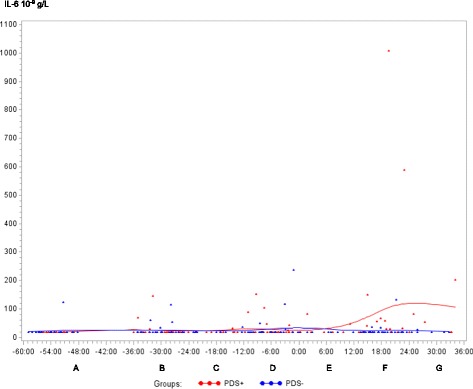
Interleukin 6 (IL-6) concentration (10^-9^ g/L) in sows with postpartum dysgalactia syndrome (PDS+, red) and healthy sows (PDS-, blue) sampled from 60 h before until 36 h after parturition (time interval A-G). Each dot represents the exact sample time of each observation relative to the exact birth of the first piglet (0 h). The lines show the mean value

**Fig. 6:**
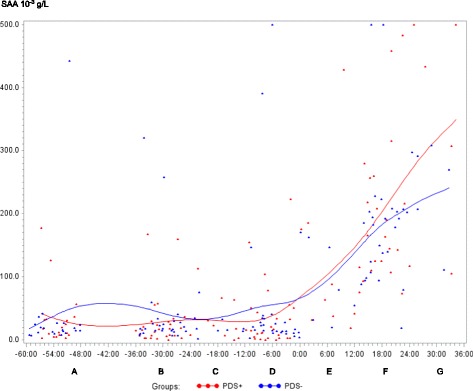
Serum amyloid A (SAA) (10^-3^ g/L) in sows with postpartum dysgalactia syndrome (PDS+, red) and healthy sows (PDS-, blue) sampled from 60 h before until 36 h after parturition (time interval A-G). Each dot represents the exact sample time of each observation relative to the exact birth of the first piglet (0 h). The lines show the mean value

**Fig. 7:**
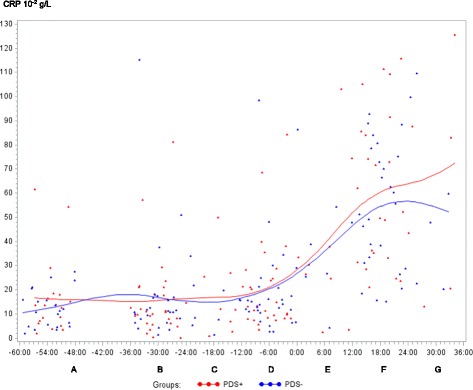
C-reactive protein (CRP) concentration (10^-2^ g/L) in sows with postpartum dysgalactia syndrome (PDS+, red) and healthy sows (PDS-, blue) sampled from 60 h before until 36 h after parturition (time interval A-G). Each dot represents the exact sample time of each observation relative to the exact birth of the first piglet (0 h). The lines show the mean value

**Fig. 8:**
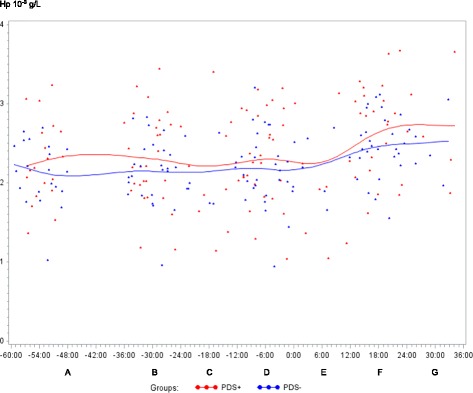
Haptoglobin (Hp) concentration (10^-3^ g/L) in sows with postpartum dysgalactia syndrome (PDS+, red) and healthy sows (PDS-, blue) sampled from 60 h before until 36 h after parturition (time interval A-G). Each dot represents the exact sample time of each observation relative to the exact birth of the first piglet (0 h). The lines show the mean value

**Fig. 9:**
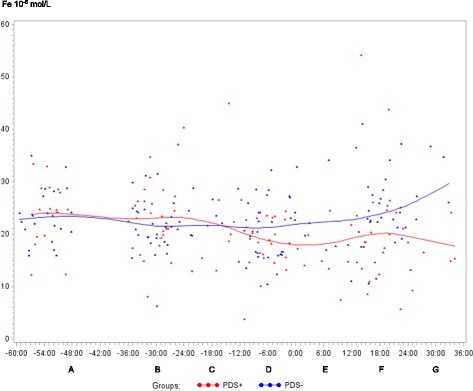
Iron (Fe) concentration (10^-6^ mol/L) in sows with postpartum dysgalactia syndrome (PDS+, red) and healthy sows (PDS-, blue) sampled from 60 h before until 36 h after parturition (time interval A-G). Each dot represents the exact sample time of each observation relative to the exact birth of the first piglet (0 h). The lines show the mean value (Normal range is 9.0 – 30.4 10^-6^ mol/L)

**Fig. 10:**
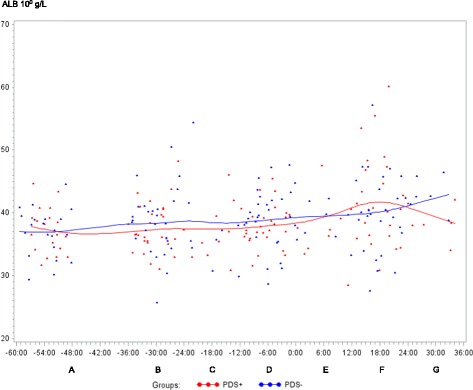
Albumin (ALB) concentration (10^0^ g/L) in sows with postpartum dysgalactia syndrome (PDS+, red) and healthy sows (PDS-, blue) sampled from 60 h before until 36 h after parturition (time interval A-G). Each dot represents the exact sample time of each observation relative to the exact birth of the first piglet (0 h). The lines show the mean value (Normal range is 32 – 48 10^0^ g/L)

In both PDS+ and PDS- sows, inflammatory markers changed in relation to parturition. Developments over time for both groups are illustrated by raw data for WBC, neutrophils, lymphocytes, TNF-α, IL-6, SAA, CRP, Hp, Fe and ALB (Figs. 1, 2, 3, 4, 5, 6, 7, 8, 9 and 10). Some markers (IL-1, TP, Hb, and Ht) did not change significantly over time in any group [See Additional file 2, Additional file 3, Additional file 4 and Additional file 5]. A leukocyte left shift was detected in one time interval in 5 PDS+ sows and 1 PDS- sow. Mild neutrophilic toxic changes were observed in one time interval in 5 PDS+ sows and 7 PDS- sows. Reactive lymphocytes in low numbers were observed in 2 PDS+ sows and in 7 PDS- sows.

**Fig. 11:**
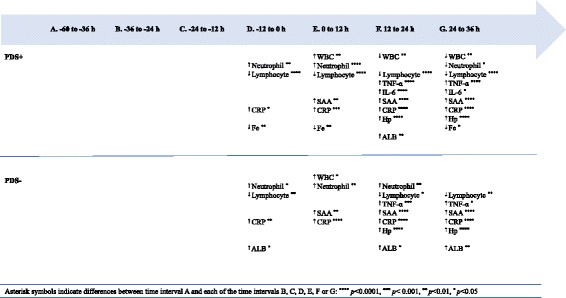
Changes in levels of inflammatory markers over time in 38 sows with postpartum dysgalactia syndrome (PDS+) and 38 healthy (PDS-) sows, with asterisk symbols indicating significant differences between time interval A and the subsequent time intervals (D to G). Upward pointing arrows indicate increasing concentrations. Downward pointing arrows indicate decreasing concentrations

Statistically significant changes of the inflammatory markers in relation to parturition for both PDS- and PDS+ sows are summarized in Fig. 11. Most inflammatory markers were changed relative to baseline (time interval A) between 12 and 36 h p.p. [WBC (Fig. 1), neutrophil counts (Fig. 2), lymphocyte counts (Fig. 3), TNF-α (Fig. 4), IL-6 (Fig. 5), SAA (Fig. 6), CRP (Fig. 7), Hp (Fig. 8), Fe (Fig. 9) and ALB (Fig. 10)], while changes in neutrophil counts (Fig. 2), lymphocyte counts (Fig. 3), CRP (Fig. 7), Fe (PDS+ sows only, Fig. 9), ALB (PDS- sows only and Fig. 10) were observed also before parturition (Fig. 11).

#### Differences between PDS+ and PDS- sows

WBC, neutrophil counts, lymphocyte counts, TNF-α, IL-6, Hp and Fe differed between PDS+ and PDS- sows (Table 2). Lymphocyte counts differed between PDS- and PDS+ sows pre-partum, with lymphocyte counts being lower in PDS+ than in PDS- sows from -12 h to 36 h. While WBC and neutrophil counts tended to be higher in PDS+ than in PDS- sows immediately around parturition (-12 to 12 h p.p.), WBC and neutrophil counts dropped in PDS+ sows and were significantly lower than in PDS- sows 12-24 h p.p. After parturition, concentrations of TNF-α, IL-6 and Hp were significantly higher in PDS+ than in PDS- sows at 12-36 h. Concentrations of Fe were significantly lower in PDS+ than in PDS- sows 24-36 h p.p. Concentrations of SAA, CRP, ALB, TP, Hb and Ht did not differ between PDS+ and PDS- sows.

**Table 2:** Least-squares means (LSMEANS) and standard deviation (SD) for white blood cell counts (WBC), neutrophil counts, lymphocyte counts, tumor necrosis factor alpha (TNF-α), interleukin 6 (IL-6), serum amyloid A (SAA), C-reactive protein (CRP), haptoglobin (Hp), iron (Fe) and albumin (ALB) in 38 sows with postpartum dysgalactia syndrome (PDS+) and 38 healthy sows (PDS-)

			A. (-60 to -36 h)		B. (-36 to -24 h)		C. (-24 to -12 h)		D. (-12 to 0 h)		E. (0 to 12 h)		F. (12 to 24 h)		G. (24 to 36 h)	
Parameter	Group	*n*	LSMEANS	±SD	LSMEANS	±SD	LSMEANS	±SD	LSMEANS	±SD	LSMEANS	±SD	LSMEANS	±SD	LSMEANS	±SD
White blood cells	PDS+	110	11.46	0.69	11.29	0.58	12.62	1.04	12.14	0.58	15.45	1.04	8.74^****^	0.66	6.15	1.45
	PDS-	126	10.77	0.58	11.22	0.57	11.68	0.97	11.09	0.55	13.94	1.16	12.20^****^	0.60	9.24	1.18
Neutrophils	PDS+	110	6.35	0.59	6.36	0.49	7.93	0.90	8.34	0.49	11.53	0.89	5.22^**^	0.56	3.35	1.25
	PDS-	126	5.57	0.49	6.39	0.49	6.43	0.83	7.09	0.48	8.9	1.00	7.46^**^	0.51	4.35	1.01
Lymphocytes	PDS+	110	3.96	1.07	3.81	1.05	3.69	1.10	2.74^*^	1.05	2.43	1.10	2.22^****^	1.06	1.90^*^	1.14
	PDS-	126	3.95	1.05	3.75	1.05	3.83	1.01	3.20^*^	1.05	3.23	1.12	3.30^****^	1.06	2.69^*^	1.12
Tumor necrosis factor-α	PDS+	121	30.04	1.15	31.81	1.13	32.27	1.21	31.02	1.13	34.35	1.21	130.43^****^	1.14	130.36^*^	1.26
	PDS-	119	31.53	1.15	32.80	1.13	29.87	1.20	31.71	1.13	34.23	1.22	55.21^****^	1.14	58.99^*^	1.25
Interleukin 6	PDS+	121	19.10	1.13	22.28	1.11	19.94	1.20	24.61	3.03	24.76	1.20	39.20^***^	1.13	39.54	1.25
	PDS-	119	20.50	1.13	22.23	1.12	21.15	1.20	22.81	1.11	20.70	1.22	22.06^***^	1.12	21.47	1.24
Serum amyloid A	PDS+	120	15.01	1.27	12.81	1.22	13.33	1.42	14.84	1.23	54.20	1.42	187.41	1.26	234.58	1.53
	PDS-	122	18.45	1.26	20.63	1.24	19.81	1.41	21.36	1.23	69.06	1.46	145.13	1.24	292.72	1.52
C-reactive protein	PDS+	122	10.03	1.18	8.71	1.13	13.72	1.26	14.86	1.16	24.60	1.26	56.30	1.17	71.52	1.32
	PDS-	122	9.23	1.18	11.80	1.16	11.05	1.25	14.65	1.16	29.14	1.27	48.52	1.17	53.02	1.31
Haptoglobin	PDS+	122	2.24	0.08	2.26	0.08	2.35	0.10	2.30	0.08	2.31	0.10	2.73^*^	0.08	2.92^*^	0.12
	PDS-	122	2.11	0.08	2.15	0.08	2.16	0.10	2.15	0.08	2.23	0.10	2.46^*^	0.08	2.56^*^	0.11
Iron	PDS+	122	24.36	1.51	23.40	1.23	22.16	2.23	18.30	1.27	16.75	2.22	20.86	1.38	17.97^*^	2.73
	PDS-	125	23.30	1.44	21.22	1.30	23.42	2.20	21.12	1.25	21.28	2.32	24.08	1.30	26.40^*^	2.68
Albumin	PDS+	122	37.31	1.09	37.38	0.90	37.82	1.60	37.74	0.93	38.38	1.59	41.99	1.00	40.15	1.95
	PDS-	125	36.67	1.04	38.52	0.94	37.17	1.57	39.73	0.91	37.79	1.66	39.88	0.95	42.83	1.91
Total protein	PDS+	122	72.29	1.03	71.20	1.02	72.76	1.04	70.74	1.02	70.73	1.04	76.14	1.03	76.50	1.05
	PDS-	125	69.30	1.03	72.54	1.02	68.87	1.04	73.29	1.02	68.92	1.04	72.93	1.02	77.71	1.05
Hemoglobin	PDS+	110	6.51	0.12	6.32	0.10	6.31	0.17	6.31	0.10	6.02	0.17	6.41	0.11	6.50	0.23
	PDS-	126	6.44	0.10	6.34	0.10	6.38	0.16	6.29	0.10	6.46	0.18	6.22	0.10	6.39	0.19
Hematocrit	PDS+	110	0.35	0.01	0.34	0.01	0.34	0.01	0.34	0.01	0.32	0.01	0.34	0.01	0.34	0.01
	PDS-	126	0.34	0.01	0.34	0.01	0.35	0.01	0.33	0.01	0.35	0.01	0.33	0.01	0.34	0.01

Asterisk symbols indicating significant differences between PDS+ and PDS- sows: ^****^ *p*<0.0001, ^***^ *p*< 0.001, ^**^ *p*<0.01, ^*^ *p*<0.05

## Discussion

### Periparturient inflammation

In the present study, hematological and blood biochemical changes indicate the presence of systemic inflammation in both healthy and PDS-affected sows. Periparturient inflammation in healthy individuals has previously been described in other species [33, 34] as well as in sows [35]. In horses, WBC [34] and SAA concentrations [36] were increased within 2 and 36 h, respectively, after foaling. In healthy primiparous sows, blood neutrophil counts increased significantly between day 3 before parturition and day 1 p.p. [37, 38], followed by a decrease back towards antepartum levels on day 3 p.p. [38]. In healthy sows, SAA and Hp concentrations were increased relative to antepartum levels 48-72 h and 24-96 h p.p., respectively [26]. Several factors may cause periparturient changes in inflammatory markers. Hormonal changes with fast antepartum cortisol increase have been suggested to be the cause of periparturient leukocytosis and neutrophilia [34]. Release of cytokines and APPs after parturition could be caused by tissue trauma to the birth canal, elicited by the foetus as well as by obstetric interventions. Understanding periparturient inflammation in otherwise healthy sows is important for differentiating physiology from pathology when using inflammatory markers for assessing periparturient disease, as inflammatory markers are non-specific in nature and will be released in infectious as well as non-infectious inflammation. Limiting periparturient inflammation may be beneficial to avoid inflammatory-induced physiological and behavioural changes such as malaise, pain, fatigue and anorexia, which may affect the sow’s ability to provide for the piglets and thus impact welfare of both sow and piglets. PDS+ as well as PDS- sows had increased serum concentrations of TNF-α and IL-6 12-36 h p.p., and these two cytokines have been shown to cause a wide range of clinical signs [39, 40]. The link between parturition-induced inflammation and disease is unexplored, but sows with larger litters and sows receiving birth interventions, and thus potentially more trauma to the birth canal, were more likely to develop coliform mastitis [14]. Pre-existing inflammation may also be a problem if animals are subsequently affected by an infectious agent. It has been shown that exposure to exogenous LPS exacerbates tissue injury and mortality in experimentally induced ischemia and reperfusion lesions [41, 42].

In sow mammary tissue, lymphocytes are located in the interalveolar tissue, in the epithelium and, during lactation within the alveolar lumen [43, 44]. At the end of gestation, the total number of lymphocytes in the mammary tissue increases but at parturition the amount decreases temporarily, followed by a second increase from the day of parturition [43, 44]. Moreover, increased opsonic activity in mammary secretion are found p.p. [38]. The observed decrease in lymphocyte counts observed between -12 and 36 h in PDS+ sows, and to some extent in PDS- sows, could therefore be explained by recruitment of lymphocytes into the mammary tissue for colostrum production. Further, Salmon [43] demonstrated a temporal association between lymphocyte accumulation and expression of prolactin-receptors on the epithelial cells which indicate that prolactin plays a role directing lymphocytes to the mammary tissue. Though, lymphocytopenia also occur in response to glucocorticoid administration in humans [44–46] and an association between increased serum cortisol and decreased numbers of mononuclear cells are found in healthy sows during parturition [37, 38]. Magnusson and Fossum [37] therefore concluded that variations in numbers of mononuclear cells could be influenced by cortisol. This could also be the case in the present study.

### Inflammatory changes in sows with PDS

The prevalence of PDS+ sows (34.9 %) in the herd indicates that high herd health and SPF status did not protect the sows from developing PDS.

Leukocyte counts (WBC, neutrophil and lymphocyte counts), serum concentrations of the proinflammatory cytokines (TNF-α and IL-6) and acute phase reactants (Hp and Fe) differed between healthy and PDS affected sows. These changes suggest that PDS+ sows suffered from more severe systemic inflammation than did the PDS- sows. The cause of this inflammation was not determined. A previous study in gilts demonstrated a decrease in WBC and neutrophils 12-36 h after LPS infusion at parturition [25] and a non-significant decrease of WBC and neutrophils was found among sows with clinical signs of agalactia day 1 and 2 p.p. [35]. The leukopenia and low neutrophil counts that developed in PDS+ sows 12-36 h p.p. could be related to overwhelming infection or exposure to LPS, as LPS may cause profound leukopenia in several species including pigs [47]. *E. coli* has been found in larger amount among agalactic sows [17], which indicate that LPS could be involved in the pathogenesis of PDS. Leukocyte left shift has been demonstrated among sows affected with porcine agalactia [48] after exposure to LPS [49] and E. coli [50], so release of this substance from the mammary gland or intestine may have resulted in the cytological changes observed in the PDS+ sows.

There was a tendency (*p*<0.1) for transient elevated neutrophil counts in PDS+ sows from -12 to 12 h compared to PDS- sows (Fig. 2). This could reflect an endocrine stress response with increased blood cortisol, as cortisol causes leukocytosis and neutrophilia by preventing migration of leukocytes to the extracellular space [51].

Concentrations of the pro-inflammatory cytokines TNF-α and IL-6 were higher in PDS+ than in PDS- sows. A previous study demonstrated higher serum TNF-α and IL-6 concentrations in sows affected with MMA compared to healthy sows for up to 72 h p.p. [24]. Experimental induction of udder inflammation by intra-mammary infusion of LPS [27] or *E. coli* inoculation [26] has been shown to cause increased blood concentrations of TNF-α and IL-6. Based on these findings, previous studies have suggested that TNF-α and IL-6 could be potential biomarkers for detection of sows with coliform mastitis or MMA [24, 26], thus corroborating the results of the present study.

Proinflammatory cytokines are responsible for the induction of hepatic synthesis of APPs. Concentrations of the three measured APPs, CRP, SAA and Hp, increased significantly peripartum in both groups of sows, but only Hp concentrations differed in PDS- and PDS+ sows. While p.p. concentrations of Hp were similar in healthy sows and sows receiving intramammary administration of *E. coli* in one study [26], another study detected higher Hp concentrations in sows with MMA than in healthy sows 1-10 days p.p. [8]. Based on our results, Hp seems to be the most useful of the three APPs measured. Considering the modest difference in Hp concentrations in PDS+ and PDS- sows (peak Hp concentration was 14 % higher in PDS+ sows than in PDS- sows), the diagnostic potential of Hp may be limited. It is not clear why concentrations of SAA, which is considered to be a very sensitive marker of inflammation [52], did not differ between the groups. In a previous study, higher blood concentrations of SAA were detected in sows undergoing intra-mammary inoculation with *E. coli* as compared to healthy controls [26]. The discrepancy between studies may be related to the timing of sampling, as different APPs have different response patterns, with some increasing more slowly than others. In the study by Zhu et al. [26] sows were sampled until 96 h p.p., and the study showed that SAA concentrations peaked at 48 h p.p. following intramammary infusion of *E. coli*, whereas concentrations of proinflammatory cytokines peaked at 24 h. To the authors knowledge, CRP has not been investigated in sows at parturition. However, CRP has well described APP properties in the porcine species, as increased concentrations have been found in pigs with clinical signs of disease [53], pigs with lesions at slaughter [54] and after experimental injection of LPS [55] or turpentine [56]. Similar to SAA, CRP could not distinguish PDS+ from PDS- sows.

Iron is a so-called negative acute phase reactant, as its concentration decreases during inflammation. In accordance with this, PDS+ sows had lower serum Fe concentrations than PDS- sows. A decrease in Fe and an increase in Hp has been shown previously in pigs following experimental inoculation with Actinobacillus [57]. Odink et al. [58] found lower serum Fe concentrations in slaughter pigs with abscesses and other inflammatory processes than in pigs with no postmortem findings. In ruminants and horses, Fe have been shown to be a fast reacting marker, with concentrations decreasing within few hours following an inflammatory stimulus, and returning to normal values within 48 h [59, 60]. In horses, assessment of serum Fe was more useful for detection of acute inflammation than was the assessment of fibrinogen [61]. The results of the present study indicate that Fe may be used for distinguishing PDS+ and PDS- sows 12-36 h p.p.

## Conclusions

This study suggests that inflammation is part of the pathogenesis in PDS. WBC, neutrophil and lymphocyte counts, and serum concentrations of TNF-α, IL6, Hp and Fe differed between PDS+ and PDS- sows and may potentially serve as diagnostic adjuncts for detection of PDS. However, considering the inflammatory changes found also in PDS- sows, the diagnostic value of measuring inflammatory markers needs further investigation in larger study populations, where diagnostic capacity (positive and negative predictive value) of the investigated markers may be fully assessed. Combining measurements of inflammatory markers with assessment of clinical and behavioral parameters may improve identification of sows at risk of developing PDS. Development of assay systems that allow farmers and veterinarians to measure relevant inflammatory markers sow-side may enhance future use of clinical-chemical parameters in porcine herd health medicine markedly. Early (antepartum) detection of sows at risk of developing PDS is desirable, but only lymphocyte counts were different in PDS+ and PDS- sows before parturition.

## Additional files

Additional file 1: Three fictive examples of sampling points. The figure illustrates how the number of observations (*n*) in each time interval differ between variables because of individual sampling times relative to parturition (0 h). (PPTX 46 kb)

Additional file 2: Interleukin 1 (IL-1). IL-1 concentration (10^-9^ g/L) in sows with postpartum dysgalactia syndrome (PDS+, red) and healthy sows (PDS-, blue) sampled from 60 h before until 36 h after parturition (time interval A-G). Each dot represents the exact sample time of each observation relative to the exact birth of the first piglet (0 h). The lines show the mean value. (DOCX 30 kb)

Additional file 3: Total protein (TP). TP concentration (10^0^ g/L) in sows with postpartum dysgalactia syndrome (PDS+, red) and healthy sows (PDS-, blue) sampled from 60 h before until 36 h after parturition (time interval A-G). Each dot represents the exact sample time of each observation relative to the exact birth of the first piglet (0 h). The lines show the mean value. (DOCX 33 kb)

Additional file 4: Hemoglobin (Hb). Hb concentration (10^-3^ mol/L) in sows with postpartum dysgalactia syndrome (PDS+, red) and healthy sows (PDS-, blue) sampled from 60 h before until 36 h after parturition (time interval A-G). Each dot represents the exact sample time of each observation relative to the exact birth of the first piglet (0 h). The lines show the mean value. Normal range is 6.2 – 9.4 × 10^-3^ mol/L. (DOCX 31 kb)

Additional file 5: Hematocrit (Ht). Ht (L/L) in sows with postpartum dysgalactia syndrome (PDS+, red) and healthy sows (PDS-, blue) sampled from 60 h before until 36 h after parturition (time interval A-G). Each dot represents the exact sample time of each observation relative to the exact birth of the first piglet (0 h). The lines show the mean value. Normal range is 0.31 – 0.46 L/L. (DOCX 41 kb)

## Abbreviations

ALB: Albumin
APP’s: Acute phase proteins
CRP: C-reactive protein
*E. coli*: *Escherichia coli*
Fe: Iron
Hb: Hemoglobin
Hp: Haptoglobin
Ht: Hematocrit
IL-1: Interleukin 1
IL-6: Interleukin 6
LPS: Lipopolysaccharide
LSMEANS: Least-squares means
MMA: Mastitis, metritis and agalactia syndrome
p.p.: Postpartum
PDS: Postpartum dysgalactia syndrome (PDS+ is cases and PDS- is healthy sows)
SAA: Serum amyloid A
SD: Standard deviation
SPF: Specific pathogen-free
TNF-α: Tumor necrosis factor alpha
TP: Total protein
WBC: White blood cell counts

## References

[1] Kemper N, Gerjets I (2009). Bacteria in milk from anterior and posterior mammary glands in sows affected and unaffected by postpartum dysgalactia syndrome (PPDS). Acta Vet Scand..

[2] Papadopoulos GA, Vanderhaeghe C, Janssens GPJ, Dewulf J, Maes DGD (2010). Risk factors associated with postpartum dysgalactia syndrome in sows. Vet J.

[3] Preissler R, Hinrichs D, Reiners K, Looft H, Kemper N (2012). Estimation of variance components for postpartum dysgalactia syndrome in sows. J Anim Breed Genet.

[4] Stiehler T, Heuwieser W, Pfuetzner A, Burfeind O (2015). The course of rectal and vaginal temperature in early postpartum sows. J Swine Health Prod.

[5] Maes D, Papadopoulos G, Cools A, Janssens GPJ (2010). Postpartum dysgalactia in sows: pathophysiology and risk factors. Tierärztl Prax.

[6] Martineau GP, Le Treut Y, Guillou D, Waret-Szkuta A (2013). Postpartum dysgalactia syndrome: a simple change in homeorhesis?. J Swine Health Prod.

[7] Quesnel H, Farmer C, Devillers N (2012). Colostrum intake. Influence on piglet performance and factors of variation. Livest Sci.

[8] Mirko CP, Bilkei G (2004). Acute phase proteins, serum cortisol and prewearing litter performance in sows suffering from periparturient disease. Acta Vet (Beograd).

[9] Persson A (1997). Mastitis in Sows. Clinical, bacteriological and cytological examinations in assessing udder health during early lactation and at weaning. Doctoral thesis. Acta Universitatis Agriculturae Sueciae. Veterinaria 10.

[10] Hales J, Moustsen VA, Nielsen MBF, Hansen CF (2013). Individual physical characteristics of neonatal piglets affect preweaning survival of piglets born in a noncrated system1. J Anim Sci..

[11] Furniss SJ (1987). Measurement of rectal temperature to predict ‘mastitis, metritis and alagactia’ (MMA) in Sows after farrowing. Prev Vet Med.

[12] Hoy S (2006). The impact of puerperal diseases in sows on their fertility and health up to next farrowing. Anim Sci.

[13] Backstrom L, Morkoc AC, Connor J, Larson R, Price W (1984). Clinical study of mastitis-metritis-agalactia in sows in Illinois. J Am Vet Med Assoc..

[14] Gerjets I, Traulsen I, Reiners K, Kemper N (2011). Assessing individual sow risk factors for coliform mastitis: a case-control study. Prev Vet Med.

[15] Göransson L (1989). The effect of dietary crude fibre content on the frequency of post partum agalactia in the sow. J Vet Med.

[16] Göransson L (1989). The effect of feed allowance in late pregnancy on the occurrence of agalactia post partum in the sow. J Vet Med.

[17] Persson A, Pedersen Mörner A, Kuhl W (1996). A long-term study on the health status and performance of sows on different feed allowances during late pregnancy. III. Escherichia coli and other bacteria, total cell content, polymorphonuclear leucocytes and pH in colostrum and milk during the first 3 weeks of lactation. Acta Vet Scand..

[18] Preissler R, Gerjets I, Reiners K, Looft H, Kemper N (2011). Prevalence of postpartun dysgalactia syndrome in sows.

[19] Preissler R, Tetens J, Reiners K, Looft H, Kemper N (2011). Biological pathway analysis of postpartum dysgalactia syndrome in sows via a genome-wide association study.

[20] Osterlundh I, Hulten F, Johannisson A, Magnusson U (2002). Sows intramammarily inoculated with Escherichia coli at parturition: I. Functional capacity of granulocytes in sows affected or non-affected by clinical mastitis. Vet Immunol Immunopathol..

[21] Gerjets I, Traulsen I, Reiners K, Kemper N (2011). Comparison of virulence gene profiles of Escherichia coli isolates from sows with coliform mastitis and healthy sows. Vet Microbiol..

[22] Hirsch AC, Philipp H, Kleemann R (2003). Investigation on the efficacy of meloxicam in sows with mastitis-metritis-agalactia syndrome. J Vet Pharmacol Ther..

[23] van Gelder KN, Bilkei G (2005). The course of acute-phase proteins and serum cortisol in mastitis metritis agalactia (MMA) of the sow and sow performance. Tijdschr Diergeneeskd..

[24] Szczubial M, Urban-Chmiel R (2008). Tumor Necrosis Factor-α and Interleukin-6 contentration in the serum of sows with the MMA syndrome. Bull Vet Inst Pulawy..

[25] Nachreiner RF, Ginther OJ (1974). Induction of agalactia by administration of endotoxin (Escherichia coli) in swine. Am J Vet Res..

[26] Zhu Y, Osterlundh I, Hulten F, Magnusson U (2004). Tumor necrosis factor-alpha, interleukin-6, serum amyloid A, haptoglobin, and cortisol concentrations in sows following intramammary inoculation of Escherichia coli. Am J Vet Res..

[27] Wang JF, Wang M, Ma JL, Jiao LG, Zhou XY, Lindberg JE (2006). The influence of intramammary lipopolysaccharide infusion on serum Ca, P, vitamin D, cytokines and cortisol concentrations in lactating sows. J Vet Med A Physiol Pathol Clin Med..

[28] Martineau GP, Farmer C, Peltoniemi O. Mammary system. In: Zimmerman JJ, Karriker LA, Ramirez A, Schwartz KJ, Stevenson GW. Diseases of swine. 10 ed. Chichester: Wiley. 2012. p. 270–93.

[29] Wright JH (1902). A rapid method for the differential staining of blood films and malarial parasites. J Med Res..

[30] Kruse R, Essen-Gustavsson B, Fossum C, Jensen-Waern M (2008). Blood concentrations of the cytokines IL-1beta, IL-6, IL-10, TNF-alpha and IFN-gamma during experimentally induced swine dysentery. Acta Vet Scand.

[31] Tecles F, Fuentes P, Martinez Subiela S, Parra MD, Munoz A, Ceron JJ (2007). Analytical validation of commercially available methods for acute phase proteins quantification in pigs. Res Vet Sci..

[32] Hernandez-Caravaca I, Gourgues SF, Rodriguez V, Estrada ED, Ceron JJ, Escribano D (2017). Serum acute phase response induced by different vaccination protocols against circovirus type 2 and Mycoplasma hyopneumoniae in piglets. Res Vet Sci..

[33] Hebisch G, Neumaier-Wagner PM, Huch R, von Mandach U (2004). Maternal serum interleukin-1 beta, -6 and -8 levels and potential determinants in pregnancy and peripartum. J Perinat Med..

[34] Nagel C, Trenk L, Aurich J, Wulf M, Aurich C (2016). Changes in blood pressure, heart rate, and blood profile in mares during the last 3 months of gestation and the peripartum period. Theriogenology..

[35] Hermansson I, Einarsson S, Ekman L, Larsson K (1978). On the agalactia post partum in the sow. A hematological and blood chemical study in affected and healthy sows. Nordisk veterinaermedicin..

[36] Coutinho da Silva MA, Canisso IF, ML MP, Johnson AE, Divers TJ (2013). Serum amyloid A concentration in healthy periparturient mares and mares with ascending placentitis. EVJ.

[37] Magnusson U, Fossum C (1990). Numerical Variations among Blood Mononuclear Cells during the Peripartal Period in the Gilt. J Vet Med Ser B.

[38] Osterlundh I, Holst H, Magnusson U (1998). Hormonal and immunological changes in blood and mammary secretion in the sow at parturition. Theriogenology..

[39] Bluthe RM, Laye S, Michaud B, Combe C, Dantzer R, Parnet P (2000). Role of interleukin-1beta and tumour necrosis factor-alpha in lipopolysaccharide-induced sickness behaviour: a study with interleukin-1 type I receptor-deficient mice. Eur J Neurosci..

[40] Harden LM, du Plessis I, Poole S, Laburn HP (2008). Interleukin (IL)-6 and IL-1 beta act synergistically within the brain to induce sickness behavior and fever in rats. Brain Behav Immun..

[41] Colletti LM, Green M (2001). Lung and liver injury following hepatic ischemia/reperfusion in the rat is increased by exogenous lipopolysaccharide which also increases hepatic TNF production in vivo and in vitro. Shock..

[42] Koike K, Moore FA, Moore EE, Poggetti RS, Tuder RM, Banerjee A (1992). Endotoxin after gut ischemia/reperfusion causes irreversible lung injury. J Surg Res..

[43] Salmon H (1987). The intestinal and mammary immune system in pigs. Vet Immunol Irnmunopathol.

[44] Slade JD, Hepburn B (1983). Prednisone-induced alterations of circulating human lymphocyte subsets. J Lab Clin Med..

[45] Fukuda R, Ichikawa Y, Takaya M, Ogawa Y, Masumoto A (1994). Circadian variations and prednisolone-induced alterations of circulating lymphocyte subsets in man. Intern Med..

[46] Fauci AS, Dale DC (1975). The effect of Hydrocortisone on the kinetics of normal human lymphocytes. Blood..

[47] Borissov I, Andonova M (2000). Escherichia coli lipopolysaccharide-induced experimental infection in piglets: clinical and laboratory findings. Revue Méd. Vét..

[48] Nachreiner RF, Ginther OJ (1972). Porcine Agalactia: Hematologic, Serum Chemical, and Clinical Changes during the Preceding Gestation. Am J Vet Res..

[49] Cort N (1986). A clinical study on the effect of a gram-negative bacterial endotoxin and cloprostenol in non-pregnant and 60-day-pregnant gilts. Anim Reprod Sci..

[50] Nachreiner RF, Garcia MC, Ginther OJ (1972). Clinical, Hematologic, and Blood Chemical Changes in Swine Given Endotoxin (Escherichia coli) During the Immediate Postpartum Period. Am J Vet Res..

[51] Elenkov IJ, Webster EL, Torpy DJ, Chrousos GP (1999). Stress, corticotropin-releasing hormone, glucocorticoids, and the immune/inflammatory response: acute and chronic effects. Ann N Y Acad Sci.

[52] Jacobsen S, Andersen PH (2007). The acute phase protein serum amyloid A (SAA) as a marker of inflammation in horses. Equine Vet Educ..

[53] Pallares FJ, Martinez-Subiela S, Seva J, Ramis G, Fuentes P, Bernabe A, Munoz A, Ceron JJ (2008). Relationship between serum acute phase protein concentrations and lesions in finishing pigs. Vet J..

[54] Gutierrez AM, Martinez-Subiela S, Ceron JJ (2015). Diagnostic accuracy of porcine acute phase proteins in meat juice for detecting disease at abattoir. Vet Rec..

[55] Escribano D, Campos PH, Gutierrez AM, Le Floc'h N, Ceron JJ, Merlot E (2014). Effect of repeated administration of lipopolysaccharide on inflammatory and stress markers in saliva of growing pigs. Vet J..

[56] Escribano D, Tvarijonaviciute A, Tecles F, Ceron JJ (2015). Serum paraoxonase type-1 activity in pigs: assay validation and evolution after an induced experimental inflammation. Vet Immunol Immunopathol..

[57] Baarsch MJ, Foss DL, Murtaugh MP (2000). Pathophysiologic correlates of acute porcine pleuropneumonia. AVMA..

[58] Odink J, Smeets JF, Visser IJ, Sandman H, Snijders JM (1990). Hematological and clinicochemical profiles of healthy swine and swine with inflammatory processes. J Anim Sci..

[59] Jacobsen S, Nielsen JV, Kjelgaard-Hansen M, Toelboell T, Fjeldborg J, Halling-Thomsen M, Martinussen T, Thoefner MB (2009). Acute phase response to surgery of varying intensity in horses: a preliminary study. Vet surg..

[60] Jacobsen S, Toelboell T, Andersen PH (2005). Dose dependency and individual variability in selected clinical, haematological and blood biochemical responses after systemic lipopolysaccharide challenge in cattle. Vet Res..

[61] Borges AS, Divers TJ, Stokol T, Mohammed OH (2007). Serum iron and plasma fibrinogen concentrations as indicators of systemic inflammatory diseases in horses. J Vet Intern Med..

