# Correction to: Inflammatory markers before and after farrowing in healthy sows and in sows affected with postpartum dysgalactia syndrome

**DOI:** 10.1186/s12917-018-1471-7

**Published:** 2018-06-01

**Authors:** M. Kaiser, M. Jacobson, P. H. Andersen, P. Bækbo, J. J. Cerón, J. Dahl, D. Escribano, S. Jacobsen

**Affiliations:** 1 Department of Veterinary Clinical Sciences, Faculty of Health and Medical Sciences, Copenhagen University, Højbakkegård Alle 5, 2630 Taastrup, Denmark; 2 Department of Clinical Sciences, Faculty of Veterinary Medicine and Animal Science, Swedish University of Agricultural Sciences, p.o. Box 7054, SE-750 07 Uppsala, Sweden; 3 SEGES, Danish Pig Research Center, Danish Agriculture & Food Council, Agro Food Park 15, 8200 Aarhus N, Denmark; 4 Department of Animal Medicine and Surgery, Regional “Campus of Excellence Mare Nostrum”, University of Murcia, 30100 Espinardo, Murcia, Spain; 5 Danish Agriculture and Food Council, Axelborg, Axeltorv 3, 1709 Copenhagen V, Denmark

## Correction

The original article [1] contains an error whereby the caption in Figure 8 is incorrect; the correct caption can be seen ahead alongside its respective image.

**Fig. 8:**
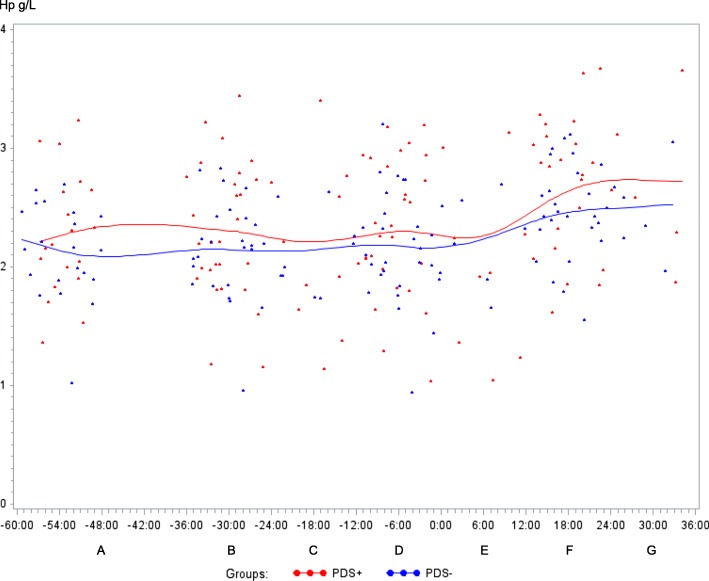
Haptoglobin (Hp) concentration (g/L) in sows with postpartum dysgalactia syndrome (PDS+, red) and healthy sows (PDS-, blue) sampled from 60 h before until 36 h after parturition (time interval A-G). Each dot represents the exact sample time of each observation relative to the exact birth of the first piglet (0 h). The lines show the mean value
