# Supplementary material for: Inflammatory markers before and after farrowing in healthy sows and in sows affected with postpartum dysgalactia syndrome
Source: BMC Vet Res. 2018 Mar 12;14:83. doi: 10.1186/s12917-018-1382-7 (PMC5848515; doi:10.1186/s12917-018-1382-7)
Supplement: Supplementary file 1 — Three fictive examples of sampling points. The figure illustrates how the number of observations (n) in each time interval differ between variables because of individual sampling times relative to parturition (0 h). (PPTX 46 kb) [file 12917_2018_1382_MOESM1_ESM.pptx]

## Slide 1
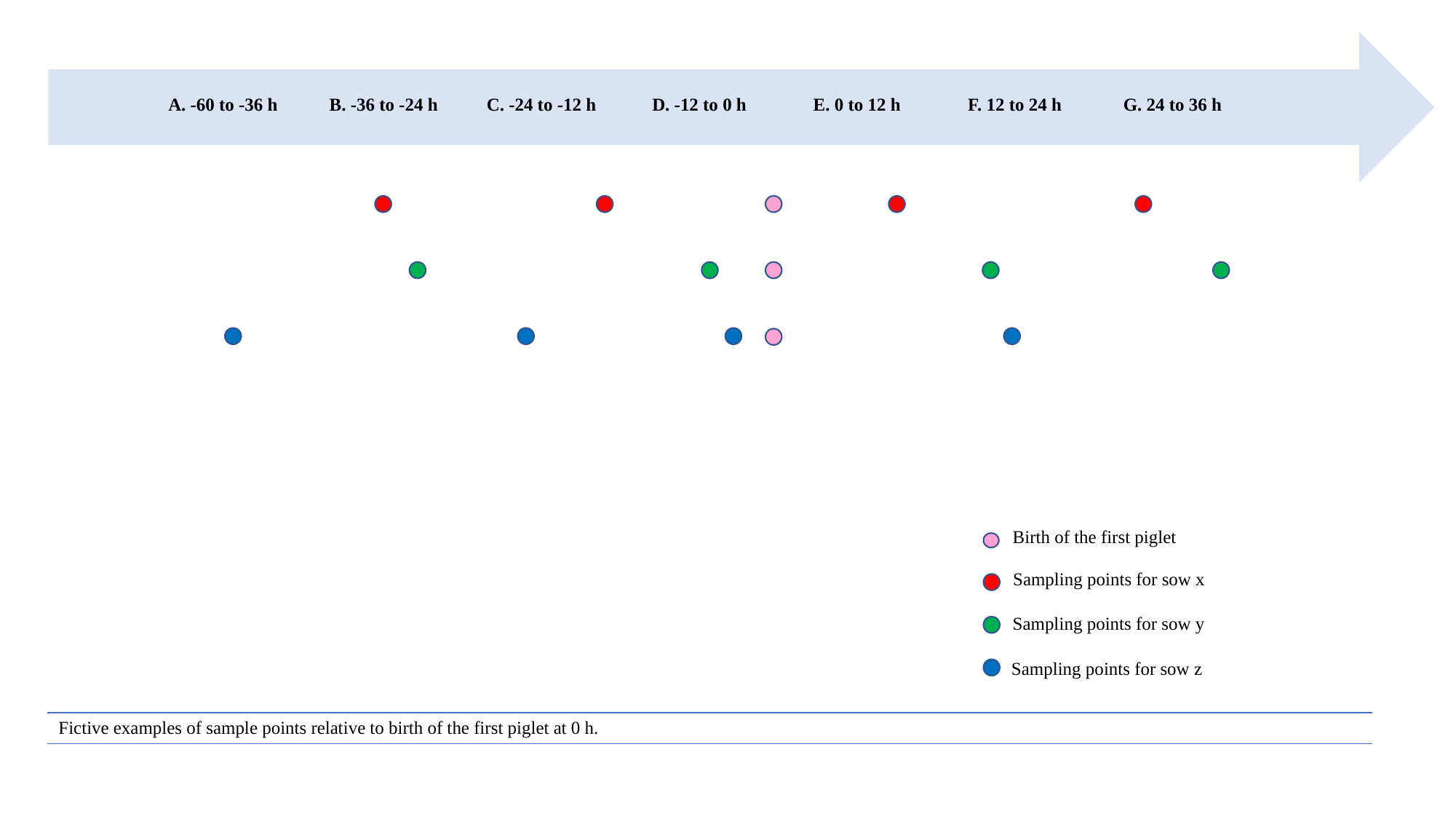

| Birth of the first piglet |
| --- |
| Sampling points for sow x |
| --- |
| Sampling points for sow y |
| --- |
| Sampling points for sow z |
| --- |
| Fictive examples of sample points relative to birth of the first piglet at 0 h. |
| --- |
