# Supplementary figures and images for: Inflammatory markers before and after farrowing in healthy sows and in sows affected with postpartum dysgalactia syndrome
Source: BMC Vet Res. 2018 Mar 12;14:83. doi: 10.1186/s12917-018-1382-7 (PMC5848515; doi:10.1186/s12917-018-1382-7)

IL-1 10-9 g/L


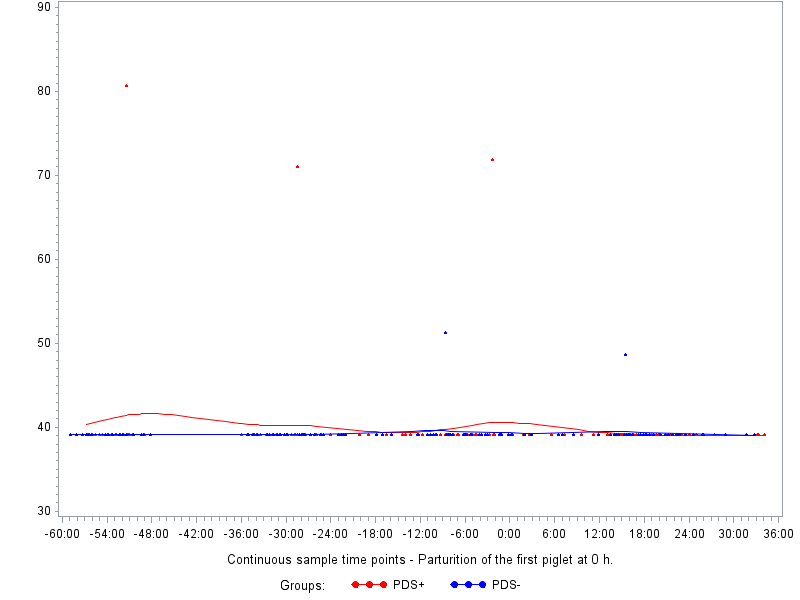


A B C D E F G

Supplement: Supplementary file 2 — Interleukin 1 (IL-1). IL-1 concentration (10-9 g/L) in sows with postpartum dysgalactia syndrome (PDS+, red) and healthy sows (PDS-, blue) sampled from 60 h before until 36 h after parturition (time interval A-G). Each dot represents the exact sample time of each observation relative to the exact birth of the first piglet (0 h). The lines show the mean value. (DOCX 30 kb) [file 12917_2018_1382_MOESM2_ESM.docx]

TP 100 g/L


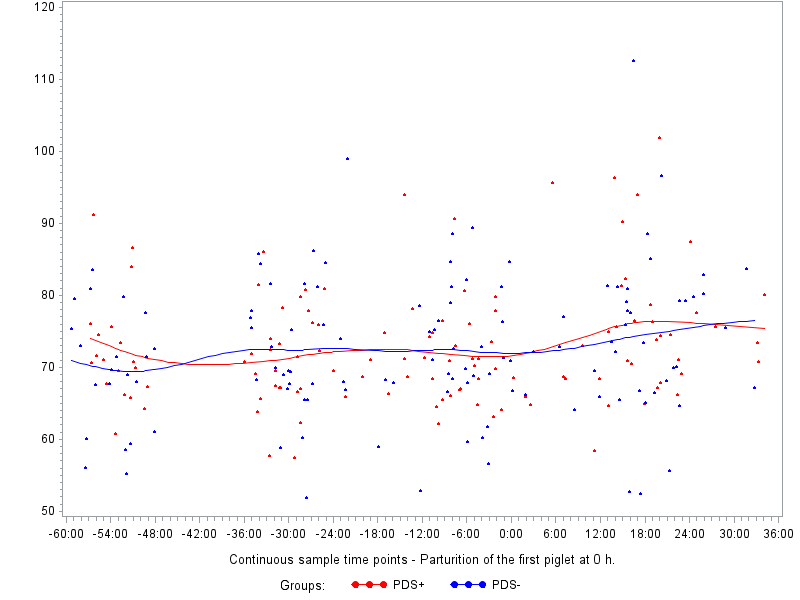


A B C D E F G

Supplement: Supplementary file 3 — Total protein (TP). TP concentration (100 g/L) in sows with postpartum dysgalactia syndrome (PDS+, red) and healthy sows (PDS-, blue) sampled from 60 h before until 36 h after parturition (time interval A-G). Each dot represents the exact sample time of each observation relative to the exact birth of the first piglet (0 h). The lines show the mean value. (DOCX 33 kb) [file 12917_2018_1382_MOESM3_ESM.docx]

Hb 10 -3 mol/L


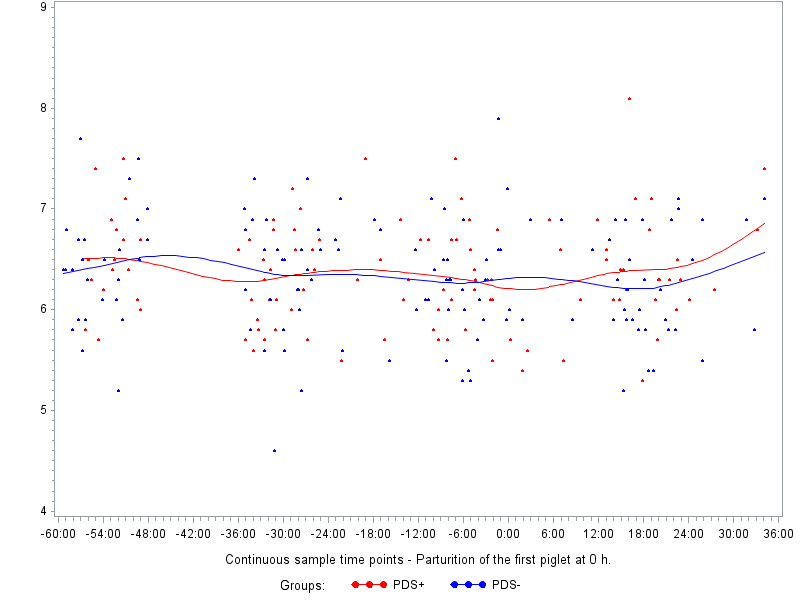


A B C D E F G

Supplement: Supplementary file 4 — Hemoglobin (Hb). Hb concentration (10-3 mol/L) in sows with postpartum dysgalactia syndrome (PDS+, red) and healthy sows (PDS-, blue) sampled from 60 h before until 36 h after parturition (time interval A-G). Each dot represents the exact sample time of each observation relative to the exact birth of the first piglet (0 h). The lines show the mean value. Normal range is 6.2 – 9.4 × 10^-3^ mol/L. (DOCX 31 kb) [file 12917_2018_1382_MOESM4_ESM.docx]

Ht L/L


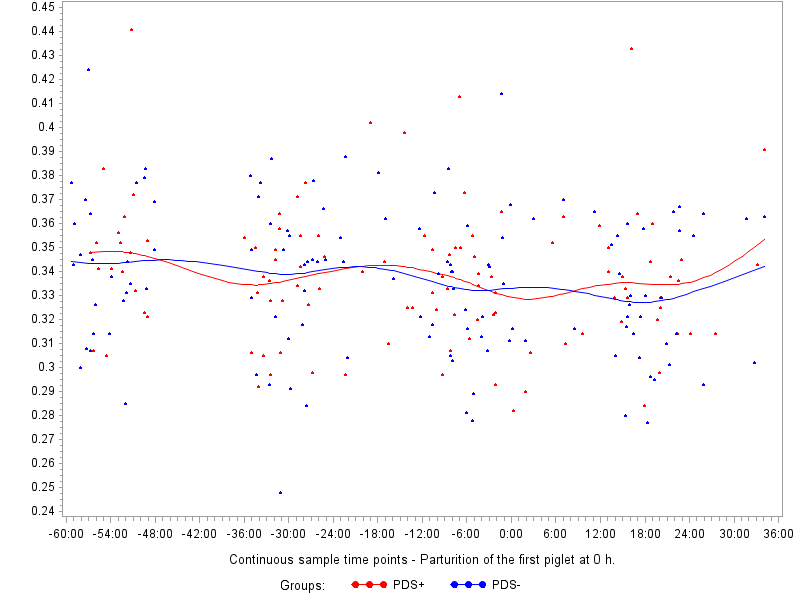


A B C D E F G

Supplement: Supplementary file 5 — Hematocrit (Ht). Ht (L/L) in sows with postpartum dysgalactia syndrome (PDS+, red) and healthy sows (PDS-, blue) sampled from 60 h before until 36 h after parturition (time interval A-G). Each dot represents the exact sample time of each observation relative to the exact birth of the first piglet (0 h). The lines show the mean value. Normal range is 0.31 – 0.46 L/L. (DOCX 41 kb) [file 12917_2018_1382_MOESM5_ESM.docx]
